# Thioredoxin targets are regulated in heterocysts of cyanobacterium *Anabaena* sp. PCC 7120 in a light-independent manner

**DOI:** 10.1093/jxb/erz561

**Published:** 2019-12-21

**Authors:** Shoko Mihara, Kazunori Sugiura, Keisuke Yoshida, Toru Hisabori

**Affiliations:** 1 Laboratory for Chemistry and Life Science, Institute of Innovative Research, Tokyo Institute of Technology, Nagatsuta-cho, Midori-ku, Yokohama, Japan; 2 School of Life Science and Technology, Tokyo Institute of Technology, Nagatsuta-cho, Midori-ku, Yokohama, Japan; 3 University of Cambridge, UK

**Keywords:** *Anabaena*, heterocyst, nitrogen fixation, oxidative pentose phosphate pathway, photosynthesis, redox regulation, thioredoxin

## Abstract

In the nitrogen-fixing cyanobacterium *Anabaena* sp. PCC 7120, glucose 6-phosphate dehydrogenase (G6PDH) plays an important role in producing the power for reducing nitrogenase under light conditions. Our previous study showed that thioredoxin suppresses G6PDH by reducing its activator protein OpcA, implying that G6PDH is inactivated under light conditions because thioredoxins are reduced by the photosynthetic electron transport system in cyanobacteria. To address how *Anabaena* sp. PCC 7120 maintains G6PDH activity even under light conditions when nitrogen fixation occurs, we investigated the redox regulation system in vegetative cells and specific nitrogen-fixing cells named heterocysts, individually. We found that thioredoxin target proteins were more oxidized in heterocysts than in vegetative cells under light conditions. Alterations in the redox regulation mechanism of heterocysts may affect the redox states of thioredoxin target proteins, including OpcA, so that G6PDH is activated in heterocysts even under light conditions.

## Teaser

In phototrophs, thioredoxin targets are generally reduced in a light-dependent manner. In contrast, thioredoxin targets in heterocysts of nitrogen-fixing cyanobacteria are regulated independently of light conditions.

## Introduction

In photosynthetic organisms, the thiol-based redox regulation system is important to control the activities of metabolic enzymes in response to fluctuating light conditions. For this regulation, a redox cascade composed of ferredoxin (Fd), Fd-thioredoxin reductase (FTR), and thioredoxin (Trx) is considered to be a major pathway for transferring reducing power from the photosynthetic electron transfer system to target proteins (Buchanan, 1980; Buchanan and Balmer, 2005). In addition, NADPH-thioredoxin reductase C (NTRC), which possesses an NADPH-thioredoxin reductase (NTR) domain and a Trx domain in one molecule, functions in the redox regulation system. NTRC reduces the target proteins using NADPH as a source of reducing power (Serrato *et al*., 2004). Trx has a conserved active site motif, WCGPC, and reduces its target proteins by a dithiol–disulfide exchange reaction. The chloroplast Trxs are classified into five subtypes: Trx-*f*, Trx-*m*, Trx-*x*, Trx-*y*, and Trx-*z* (Serrato *et al*., 2013). Four Calvin–Benson cycle enzymes, glyceraldehyde 3-phosphate dehydrogenase, fructose 1,6-bisphosphatase, sedoheptulose 1,7-bisphosphatase, and phosphoribulokinase, the γ subunit of ATP synthase, malate dehydrogenase, and glucose 6-phosphate dehydrogenase (G6PDH), the first enzyme of the oxidative pentose phosphate pathway (OPPP), are well-studied Trx target proteins in chloroplasts (Buchanan and Wolosiuk, 1976; Buchanan, 1980; Mills *et al*., 1980; Scheibe and Anderson, 1981). Although the four Calvin–Benson cycle enzymes, ATP synthase, and malate dehydrogenase are activated when reduced by Trx under light conditions, G6PDH is inactivated by reduction. This opposite regulation is reasonable to prevent the release of fixed carbon by photosynthesis.

Although cyanobacteria are thought to be the ancestral organisms from which the current chloroplasts in plants are derived, their redox regulation system is apparently different from that in chloroplasts because the compositions of Trx reductases, Trxs, and target proteins are not identical. In addition, three Trx subtypes, Trx-*m*, Trx-*x*, and Trx-*y*, are conserved within some cyanobacteria and chloroplasts, whereas Trx-*f* and Trx-*z* are not found in cyanobacteria (Florencio *et al*., 2006). Furthermore, some cyanobacteria possess different Trx reductases, including NTR and deeply rooted bacterial thioredoxin reductase (DTR), and other types of Trxs such as TrxC (Florencio *et al*., 2006, Buey *et al*., 2017). In cyanobacteria, G6PDH was also reported to be a redox-regulated enzyme, although its regulation mechanism is completely different from that in plant chloroplasts. Recently, we have reported that cyanobacterial G6PDH activity is regulated via the change in redox states of its activator protein OpcA (Mihara *et al*., 2018).

In the wild, some cyanobacteria can directly fix atmospheric nitrogen into ammonia in the cell. Because the nitrogenase complex working for this process is easily inactivated by molecular oxygen, nitrogen-fixing cyanobacteria spatially or temporally separate the nitrogen fixation system from oxygenic photosynthetic activity. The unicellular cyanobacterium *Cyanothece* sp. ATCC 51142 fixes nitrogen at night, whereas the filamentous cyanobacterium *Anabaena* sp. PCC 7120 (*Anabaena* 7120) fixes nitrogen during the day (Colon-Lopez *et al*., 1997; Toepel *et al*., 2008). To protect nitrogenase complexes from attack by photosynthetically evolved molecular oxygen, *Anabaena* 7120 differentiates its vegetative cells into specialized cells named heterocysts under nitrogen-depleted conditions. The heterocyst cells containing the nitrogenase complex are surrounded by a thick cell wall to avoid oxygen invasion. In addition, heterocysts lack PSII and show increased respiration to maintain a microoxic environment (Nicolaisen *et al*., 2009; Kumar *et al*., 2010). Moreover, heterocysts lack a carbon assimilation system, and therefore carbon is supplied from adjacent vegetative cells (Cumino *et al*., 2007; Kumar *et al*., 2010). The carbon source is then catabolized by OPPP in heterocysts, and NADPH is produced. NADPH is used as a source of power to reduce nitrogenase.

G6PDH is the initial enzyme of the OPPP in heterocyst-forming, nitrogen-fixing cyanobacteria. A recent study has shown that G6PDH accompanies the accessory protein OpcA, which is the redox-regulated protein and is reduced by Trx (Mihara *et al*., 2018). When OpcA is reduced, G6PDH is inactivated in the cell. When nitrate is present in the medium, OpcA in *Anabaena* 7120 cells is reduced under light conditions (Mihara *et al*., 2018). In contrast, more than half of OpcA was oxidized in the absence of a combined nitrogen source, even under light conditions. Consequently, G6PDH activity was maintained under such conditions. This unique regulation system enables *Anabaena* 7120 to fix nitrogen even under light conditions. However, this light-independent redox regulation mechanism of G6PDH via OpcA remains unclear. In this study, we report the differences in the redox regulation system between vegetative cells and heterocysts in *Anabaena* 7120.

## Materials and methods

### Expression and purification of recombinant proteins and activity assay

Primers to amplify the genes of *Anabaena* 7120 were designed using genome data from CyanoBase (Fujisawa *et al*., 2017). The plasmids of Trx-*m*1, Trx-*m*2, Trx-*m*3, Trx-*x*, Trx-*y*, and NTRC were constructed as described previously (Mihara et al., 2017, 2018). DNA fragments encoding NTR (alr2204), ferredoxin-NADP^+^ reductase (FNR) (all4121), and Alr2205 were cloned into the pET-23c expression vector (Novagen). The NTR plasmid was designed to express the protein with a His-tag at the C-terminus. The plasmid of the FTR heterodimer was constructed as described previously (Yoshida and Hisabori, 2017). Amplified DNA fragments encoding the FTR catalytic subunit (FTR-C) (alr4065) and the FTR variable subunit (FTR-V) (asl2469) were cloned into the *Nco*I/*Eco*RI and *Nde*I/*Xho*I sites of the pETDuet-1 vector (Novagen), respectively. These plasmids were transformed into *Escherichia coli* strain BL21 (DE3). Culture, induction of protein expression, and protein purification were performed as described previously (Mihara *et al*., 2017). His-tagged NTR and NTRC were purified by Ni-nitrilotriacetic acid affinity chromatography. Trxs and FNR were purified by anion exchange chromatography and hydrophobic interaction chromatography. The FTR heterodimer was purified using anion exchange chromatography, hydrophobic interaction chromatography, and gel filtration chromatography, as described previously (Yoshida and Hisabori, 2017). Purified proteins were concentrated using an Amicon Ultra filter (Merck Millipore), and protein concentrations were determined using BCA protein assays (Thermo Fisher Scientific), with the exception of FTR. For measurement of the protein concentrations of FTR, the Bradford assay was used (Bio-Rad). BSA was used as a standard. The NADPH-dependent 5,5'-dithiobis (2-nitrobenzoic acid) (DTNB) reduction activity of NTR was measured as described previously (Yoshida and Hisabori, 2016).

### Trx reduction by Trx reductase

Each Trx (25 µM) was treated with 25 µM diamide for 30 min at 30 °C and dialyzed before reaction with Trx reductases. To analyze FTR-dependent Trx reduction, 2 µM oxidized Trxs were incubated with 0.5 mM NADPH, 0.2 µM FNR, 1 µM Fd from *Spinacia oleracea* (Sigma-Aldrich), and 1 µM FTR in 50 mM Tris–HCl (pH 7.5) containing 50 mM NaCl. To analyze NTR- or NTRC-dependent Trx reduction, 2 µM oxidized Trxs were incubated with 0.5 mM NADPH and 1 µM NTR or NTRC in 50 mM Tris–HCl (pH 7.5) containing 50 mM NaCl. After incubation for 30 min at 30 °C, proteins were precipitated with 10% (w/v) trichloroacetic acid (TCA) and washed with ice-cold acetone. Precipitates were then suspended in SDS sample buffer [62.5 mM Tris–HCl (pH 6.8), 2% (w/v) SDS, 7.5% (v/v) glycerol, and 0.01% (w/v) bromophenol blue] containing 2 mM of the thiol-modifying reagent 4-acetamido-4′-maleimidylstilbene-2,2′-disulfonate (AMS). After modifying the free thiols of proteins with AMS for 30 min at room temperature, protein samples were subjected to non-reducing SDS–PAGE.

### Determination of the midpoint redox potential values of Trxs

Each recombinant protein (1 µM) was incubated in 25 mM Tris–HCl (pH 7.0) containing 50 mM oxidized DTT and various concentrations of reduced DTT. After incubation for 3 h at 25 °C, proteins were precipitated with 10% (w/v) TCA and washed with ice-cold acetone. Precipitates were then suspended in SDS sample buffer containing 2 mM AMS. After modifying free thiols with AMS for 1 h at room temperature, protein samples were subjected to non-reducing SDS–PAGE. The reduction levels of Trxs were then plotted against redox potentials calculated by the ratio of reduced DTT and oxidized DTT. A value of −327 mV was used as the midpoint redox potential (*E*_m_) of DTT at pH 7.0. The *E*_m_ value of each Trx was calculated by fitting the titration data to the Nernst equation.

### Construction of the strain expressing green fluorescent protein (GFP)-tagged CP12ΔC

The truncated C-terminal region (CP12ΔC) (27 amino acids truncated at the C-terminal end) of CP12 (asl2850) was used as the Trx target peptide. The plasmids were constructed by the Hot Fusion method (Fu *et al*., 2014). *luxAB* was removed from pRL502 (Elhai, 1993) (pRL502Δ*luxAB*), and pRL502Δ*luxAB* was used as a vector (Higo *et al*., 2016). The *nifB* promoter (*PnifB*) was used for heterocyst-specific expression, and the *rbcL* promoter (*PrbcL*) was used for vegetative cell-specific expression of GFP-tagged CP12ΔC. Each of the promoters, *cp12ΔC* and *gfpmut2* (Cormack *et al*., 1996), was cloned into the *Eco*RI/*Bam*HI sites of pRL502Δ*luxAB*. The resulting plasmids were transferred by conjugation into *Anabaena* 7120, as described previously (Elhai and Wolk, 1988), and introduction of the plasmid into the cell was confirmed using PCR.

### Bacterial strains and growth conditions

The *trxC*-knockout mutant was constructed as described previously (Deschoenmaeker *et al*., 2018). *Anabaena* 7120, GFP-tagged CP12ΔC-expressing strains, and *trxC*-knockout mutant cells were grown in BG-11 medium (Rippka *et al*., 1979) or BG-11_0_ medium (nitrogen-free medium) supplemented with 20 mM HEPES–NaOH (pH 7.5). Cells were grown at 30 °C under continuous low light (30 µmol photons m^−2^ s^−1^) or high light (200 µmol photons m^−2^ s^−1^) conditions. Cultures were bubbled with air containing 1% (v/v) CO_2_.

### Enrichment of heterocysts

The enrichment of heterocysts from whole filaments was performed as described previously (Golden *et al*., 1991; Watanabe *et al*., 2014). The purity of heterocysts was checked by microscopy and immunoblotting using NifH polyclonal antibody raised against the recombinant NifH from *Anabaena* 7120 and RbcL polyclonal antibody (Agrisera, AS07-218).

### Analysis of *in vivo* redox states of proteins

*In vivo* redox states of redox proteins were analyzed using immunoblotting, as described previously (Mihara *et al*., 2018). Trx-*m* and FTR-C antibodies raised against the recombinant protein of *Synechocystis* sp. PCC 6803, polyclonal antibodies raised against recombinant Trx-*m*2, Trx-*m*3, Trx-*x*, Trx-*y*, TrxC, Alr2205, NTR, NTRC, and OpcA proteins of *Anabaena* 7120, GFP polyclonal antibody (Sigma, G1544), and GFP polyclonal antibody (Abcam, ab290) were used. The chemiluminescence of the horseradish peroxidase-conjugated secondary antibody was detected using a LAS 3000 instrument (Fuji Film, Tokyo, Japan).

## Results

### Electron flow from Trx reductases to Trxs

**Fig. 1.**
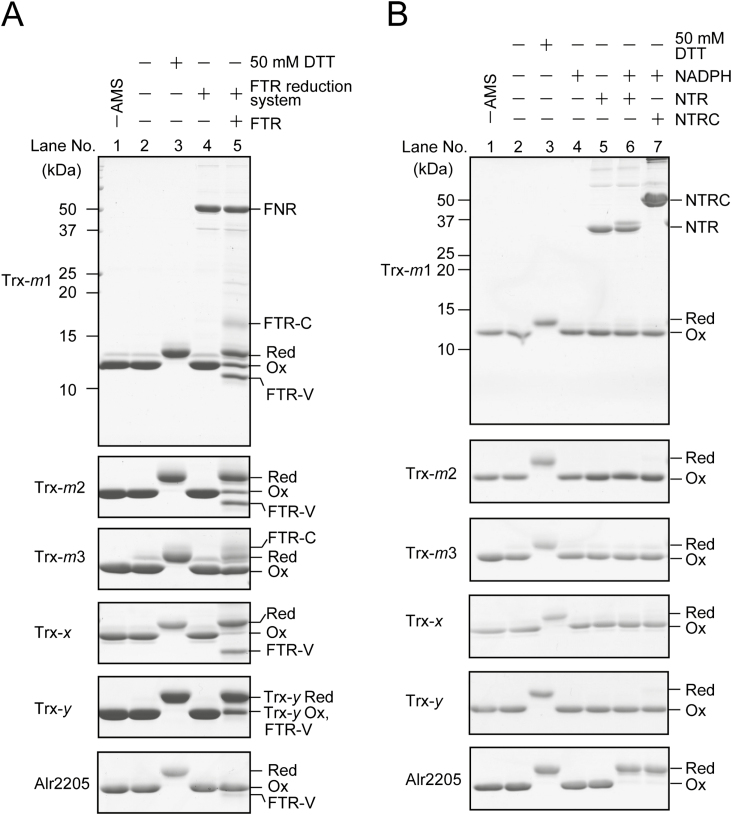
Trx reduction by FTR (A), NTR, or NTRC (B). The oxidized Trxs (2 µM) were incubated with 0.5 mM NADPH, 0.2 µM FNR, 1 µM Fd, and 1 µM FTR for FTR-dependent reduction. The mixture of NADPH, FNR, and Fd was designated as an FTR reduction system. The oxidized Trxs (2 µM) were incubated with 0.5 mM NADPH and 1 µM NTR or NTRC. Proteins were precipitated with 10% (w/v) TCA, resuspended in SDS sample buffer containing 2 mM AMS, subjected to non-reducing SDS–PAGE, and stained with Coomassie brilliant blue. Red, reduced form; Ox, oxidized form.

In the *Anabaena* 7120 genome, eight genes have been categorized as Trx-encoding genes (Trx-*m*1, Trx-*m*2, Trx-*m*3, Trx-*x*, Trx-*y*, TrxC, Alr2205, and Asl7641) in addition to the genes for FTR, NTR, and NTRC (Florencio *et al*., 2006). The phylogenetic tree of Trx is shown in Supplementary Fig. S1 at *JXB* online. Although FTR reduces Trx in a light-dependent manner, NTR and NTRC may have the ability to reduce Trx proteins irrespective of the light conditions. To clarify whether Trxs are reduced by NTR or NTRC, we examined the transfer of reducing power from FTR, NTR, or NTRC to Trx proteins. First, we expressed each of the recombinant proteins of FTR, FNR, NTR, NTRC, Trx-*m*1, Trx-*m*2, Trx-*m*3, Trx-*x*, Trx-*y*, and Alr2205 from *Anabaena* 7120 in *E. coli* and purified them. Regarding NTR, its activity was measured by the reduction of DTNB. NTR showed NADPH-dependent DTNB reduction identical to typical NTR (Supplementary Fig. S2). We oxidized Trx proteins using diamide and subsequently removed excess diamide by dialysis. The oxidized Trx proteins were then incubated with FTR, NTR, or NTRC in the presence of the FTR reduction system or NADPH. The redox states of Trx proteins were examined using the thiol-modifying reagent AMS followed by non-reducing SDS–PAGE. All Trx proteins except for Alr2205 were reduced by FTR, but not by NTR or NTRC (Fig. 1A, lane 5; Fig. 1B, lanes 6 and 7). In contrast, Alr2205 was specifically reduced by NTR and NTRC but not by FTR (Fig. 1A, lane 5; Fig. 1B, lanes 6 and 7). These results imply that most of the *Anabaena* Trxs are reduced in a light-dependent manner. Although most Trxs showed clear responses under our experimental conditions, Trx-*m*3 did not show significant changes in the redox state (Fig. 1A, lane 5).

**Fig. 2.**
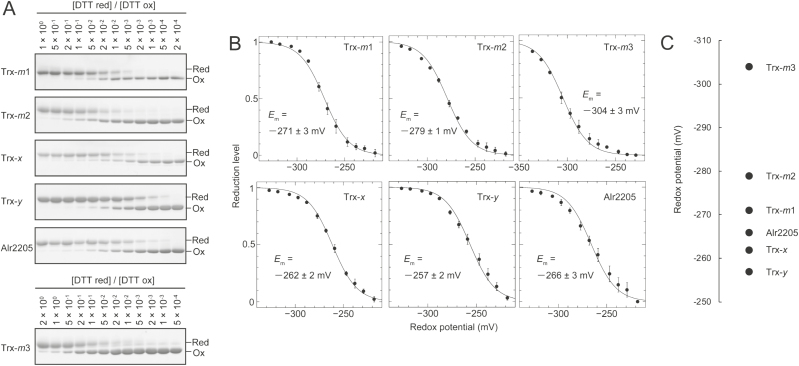
Determination of the *E*_m_ values of Trxs. (A) Trxs were equilibrated with redox buffer containing various concentrations of reduced DTT and 50 mM oxidized DTT. Proteins were precipitated with 10% (w/v) TCA, resuspended in SDS sample buffer containing 2 mM AMS, loaded on non-reducing SDS–PAGE gels, and stained with Coomassie brilliant blue. Red, reduced form; Ox, oxidized form. (B) The reduction levels of Trxs were plotted against the redox potential of DTT buffer. The *E*_m_ values of Trxs were calculated by fitting the data to the Nernst equation. These values are presented as means ±SD (*n*=3). (C) The *E*_m_ values of Trxs were compared.

**Fig. 3.**
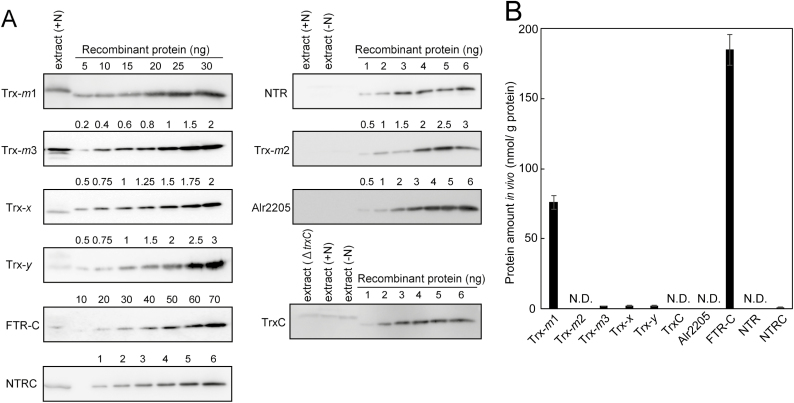
Estimation of the protein levels of Trxs and Trx reductases in *Anabaena* 7120. (A) Dilution series of recombinant proteins and the proteins extracted from *Anabaena* 7120 grown in the presence of nitrate (+N) or in the absence of combined nitrogen (–N) were subjected to SDS–PAGE and detected by immunoblotting. (B) *In vivo* levels of proteins were estimated by image analysis. Data are presented as means ±SD (*n*=3). N.D., not detected.

The *E*_m_ values of Trx proteins were then determined. The *E*_m_ value of Trx-*m*3 was more negative than those of the other Trxs (Fig. 2A–C). This result is consistent with the finding that Trx-*m*3 was hardly reduced by FTR (Fig. 1A). Although NTR and NTRC only reduced Alr2205, this protein did not show a unique *E*_m_ value. These results imply that FTR, NTR, and NTRC can reduce Trxs irrespective of the *E*_m_ values of Trx proteins. We next estimated the protein expression levels of Trxs and Trx reductases in *Anabaena* 7120 cultured in the presence or absence of nitrate by using the antibodies for these proteins. The entire blot images are shown in Supplementary Fig. S3. As shown in Fig. 3A and B, the amount of Trx-*m*1 was remarkably higher than that of other isoforms. Trx-*m*2, TrxC, Alr2205, and NTR were not detected under our experimental conditions (Fig. 3A). When immunoblotting was performed using anti-TrxC antibodies, a single band was detected, irrespective of the nitrogen conditions (Fig. 3A). However, this band was also detected in the *trxC*-deficient mutant, indicating that this is a non-specific band (Fig. 3A). In the case of Trx-*m*1, Trx-*m*3, Trx-*x*, Trx-*y*, and NTRC, we confirmed that each detected band had disappeared in each knockout mutant (Supplementary Fig. S4).

### *In vivo* redox states of Trxs under different light or nitrogen conditions

**Fig. 4.**
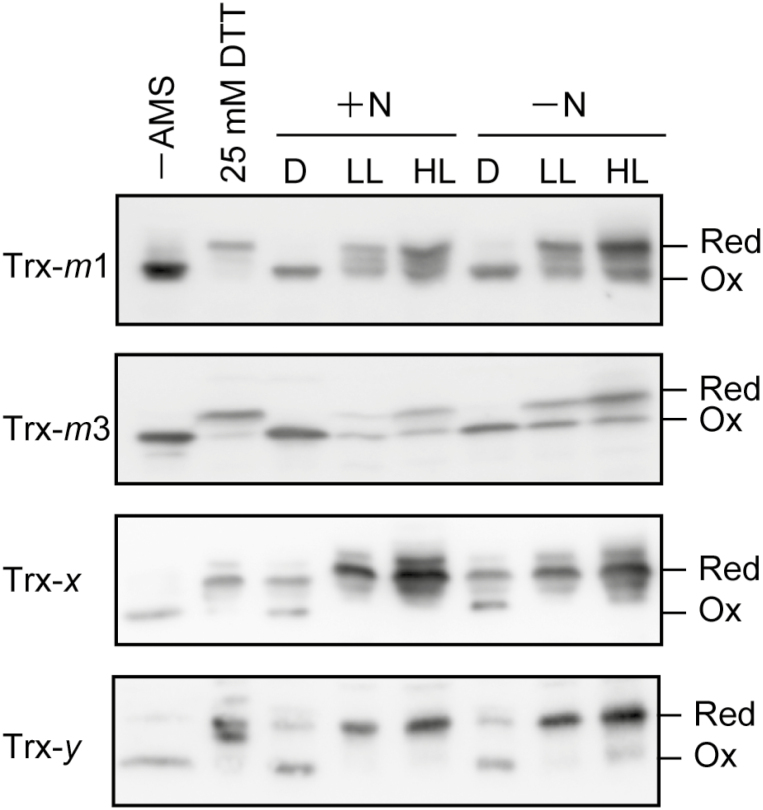
Redox states of Trxs in *Anabaena* 7120. Proteins were extracted from *Anabaena* 7120 grown in the presence of nitrate (+N) or in the absence of combined nitrogen (–N) under dark (D), low light (30 µmol photons m^−2^ s^−1^; LL), or high light (200 µmol photons m^−2^ s^−1^; HL) conditions. For the preparation of reduced proteins, cells were treated with 25 mM DTT for 10 min before adding TCA. *In vivo* redox states of Trxs were analyzed as described in the Materials and methods. Red, reduced form; Ox, oxidized form. Protein samples (20–40 µg) were loaded in each lane.

These biochemical analyses suggested that the redox state of each of the Trx isoforms in *Anabaena* 7120 changes in response to light conditions. We therefore examined whether the nitrogen source affects the *in vivo* redox states of Trxs. Proteins were extracted from *Anabaena* 7120 cultured in the presence or absence of 17.6 mM NaNO_3_ under low (30 µmol photons m^−2^ s^−1^) or high light conditions (200 µmol photons m^−2^ s^−1^). To investigate the redox states of Trx isoforms under dark conditions, the cells were kept in the dark for 3 h. Free thiols of proteins were then labeled with AMS, and the redox states of Trxs were examined using non-reducing SDS–PAGE and immunoblotting. Nitrogen deprivation did not significantly affect the redox states of Trxs (Fig. 4). Trx-*m*1 and Trx-*m*3 were detected as the oxidized form under dark conditions. In contrast, Trx-*x* and Trx-*y* were partially reduced (Fig. 4). In the case of Trx-*m*1 and Trx-*m*3, both oxidized and reduced forms were detected under low and high light conditions (Fig. 4). In contrast, Trx-*x* and Trx-*y* were mostly observed as the reduced form irrespective of the light conditions (Fig. 4).

### Redox states of Trx target protein in vegetative cells and heterocysts

**Fig. 5.**
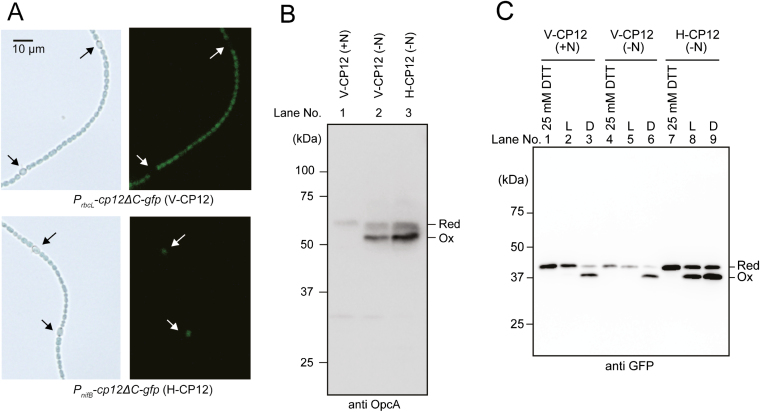
Redox states of Trx target protein in vegetative cells and heterocysts. (A) Protein expression of GFP-tagged CP12ΔC in the V-CP12 or H-CP12 strain was observed under a fluorescence microscope. Heterocysts are indicated by arrows. (B) *In vivo* redox states of OpcA in V-CP12 and H-CP12 strains were analyzed. Proteins were extracted from a 3 d culture of the V-CP12 or H-CP12 strain grown in the presence of nitrate (+N) or in the absence of combined nitrogen (–N) under light conditions (30 µmol photons m^−2^ s^−1^). Red, reduced form; Ox, oxidized form. Protein samples (20 µg) were loaded per lane. (C) *In vivo* redox states of GFP-tagged CP12ΔC in V-CP12 and H-CP12 strains were analyzed. Proteins were extracted from a 3 d culture of V-CP12 or H-CP12 strains grown in the presence of nitrate (+N) or in the absence of combined nitrogen (–N) under dark (D) or light (L) conditions. The proteins extracted from DTT-treated cells were also loaded. Red, reduced form; Ox, oxidized form.

Our *in vitro* and *in vivo* analyses indicated that the reduction of Trxs occurs in a light-dependent manner in *Anabaena* 7120. However, a previous report showed that the Trx-*m*1 target protein OpcA is mostly oxidized in the absence of nitrate, even under light conditions (Mihara *et al*., 2018). Because the oxidized form of OpcA plays an important role in the production of power for reducing nitrogenase, we assumed that OpcA is reduced in vegetative cells but oxidized in heterocysts under light conditions. We therefore prepared GFP-tagged CP12ΔC to investigate the redox states of Trx target proteins in vegetative cells and heterocysts. CP12ΔC was successfully used as a core protein for the redox sensor (Sugiura *et al*., 2019). GFP-tagged CP12ΔC was expressed under the control of the *rbcL* promoter or the *nifB* promoter in *Anabaena* 7120. The strains expressing GFP-tagged CP12ΔC in vegetative cells or heterocysts were designated as V-CP12 and H-CP12, respectively. We confirmed their expression in each cell by monitoring GFP fluorescence (Fig. 5A). We first examined the *in vivo* redox states of OpcA to check whether the overexpression of GFP-tagged CP12ΔC affects intracellular redox states. OpcA was detected as a reduced form in the presence of nitrate, but was mostly oxidized in the absence of combined nitrogen under light conditions in both the V-CP12 and H-CP12 strains, which indicates that the redox states of OpcA in these strains are similar to that of the wild type (Fig. 5B). This implies that the overexpression of GFP-tagged CP12ΔC does not markedly affect the redox states of Trx target proteins. We therefore examined the redox states of GFP-tagged CP12ΔC in the strains V-CP12 and H-CP12 cultivated under different nitrogen conditions. In the V-CP12 strain, GFP-tagged CP12ΔC was detected as the reduced form irrespective of nitrogen conditions in the light (Fig. 5C, lanes 2 and 5). In contrast, more than half of GFP-tagged CP12ΔC was detected as the oxidized form in the strain H-CP12 (Fig. 5C, lane 8). These results imply that Trx target proteins are more oxidized in heterocysts than in vegetative cells under light conditions.

**Fig. 6.**
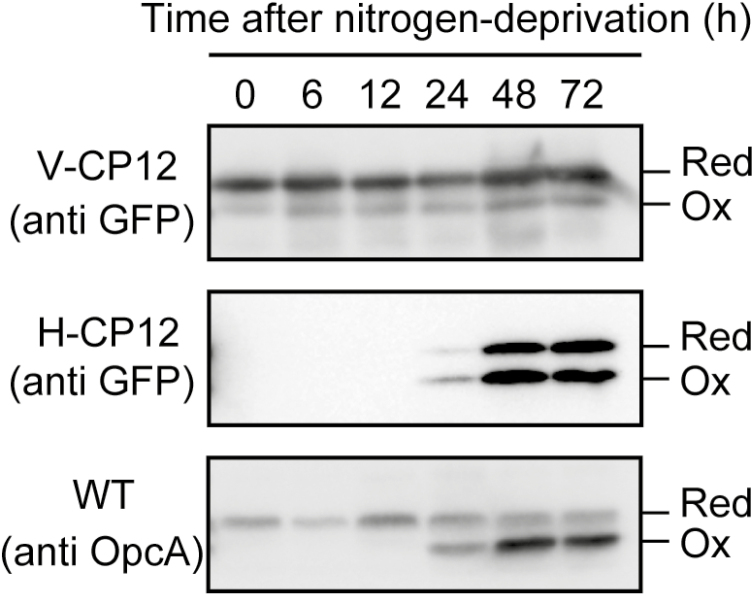
Time-course analysis of *in vivo* redox states of OpcA or GFP-tagged CP12ΔC in response to nitrogen deprivation. Proteins were extracted from *Anabaena* 7120, V-CP12, or H-CP12 strains grown in the absence of combined nitrogen under light conditions (30 µmol photons m^−2^ s^−1^). Red, reduced form; Ox, oxidized form. Protein samples (20–40 µg) were loaded in each lane.

We next investigated the transition of the redox states of GFP-tagged CP12ΔC and OpcA after nitrogen deprivation. GFP-tagged CP12ΔC in the H-CP12 strain was detected at 24 h after nitrogen deprivation, and both the oxidized and reduced forms were maintained (Fig. 6). In contrast, GFP-tagged CP12ΔC in the V-CP12 strain was fully reduced, and the status was not affected by nitrogen deprivation (Fig. 6). These results indicate that the redox states of Trx target proteins are maintained in vegetative cells during heterocyst development. We simultaneously examined the redox state of OpcA in whole filaments. OpcA was present as the reduced form by 12 h after nitrogen deprivation, but more than half of OpcA was present as the oxidized form at 24 h after nitrogen deprivation (Fig. 6). This implies that the amounts of OpcA and their redox states varied in accordance with heterocyst formation.

### Protein expression levels of Trx and FTR in heterocysts

**Fig. 7.**
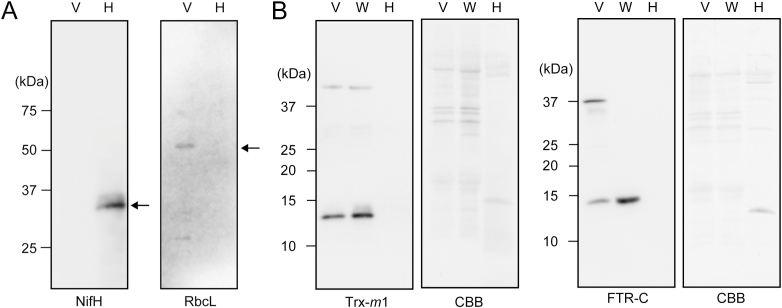
Western blot analysis of Trx-*m*1 and FTR-C in vegetative cells (V), whole cells (W), and heterocysts (H). (A) The purity of heterocysts was checked by immunoblotting using RbcL and NifH antibodies. Proteins extracted from vegetative cells and enriched heterocysts (1 µg) were loaded in each lane. (B) Trx-*m*1 and FTR-C were detected using each specific antibody. Protein extracts (20 µg) from vegetative cells, whole cells, or heterocysts were loaded per lane.

We assumed that differences in the composition of redox regulation factors may result in differences in the redox states of Trx target proteins between vegetative cells and heterocysts. We examined the protein expression levels of FTR and Trx-*m*1, an efficient electron donor for OpcA, in heterocysts. Heterocysts were isolated from whole *Anabaena* filaments cultured for 3 d or 4 d under nitrogen-depleted conditions. Proteins were then extracted from enriched heterocysts or cells grown in the presence or absence of combined nitrogen. The purity of heterocysts was confirmed by microscopy and immunoblotting using anti-RbcL antibodies and anti-NifH antibodies. RbcL was not detected, but NifH was detected in heterocyst fractions (Fig. 7A). As shown in Fig. 7B, Trx-*m*1 and FTR were detected in the vegetative cell fraction and whole-cell fraction, but not in the heterocyst fraction, indicating the drastic decrease of their protein expression in heterocysts.

## Discussion

G6PDH is important for providing power for reducing nitrogenase in nitrogen-fixing cyanobacteria. In the nitrogen-fixing, heterocyst-forming cyanobacterium *Anabaena* 7210, G6PDH activity is suppressed when its activator protein OpcA is reduced by Trx-*m*1 (Mihara *et al*., 2018). In a previous report, it was shown that OpcA was mostly oxidized to maintain G6PDH activity even under light conditions when the cells were cultured in the absence of combined nitrogen (Mihara *et al*., 2018). However, this light-independent regulatory mechanism of OpcA has not been elucidated. In this study, we found that Trx reduces target proteins in response to light conditions (Figs 1A, 4), but Trx target proteins are oxidized in heterocysts even under light conditions (Fig. 5C, lane 8).

Although a number of cyanobacteria have only one *m*-type Trx, nitrogen-fixing, heterocyst-forming cyanobacteria such as *Anabaena* 7120 and *Nostoc punctiforme* ATCC 29133 have three *m*-type Trxs designated as Trx-*m*1, Trx-*m*2, and Trx-*m*3. Because previous reports have shown that Trx-*m*2 and Trx-*m*3 could not compensate for the defect of Trx-*m*1 (Mihara *et al*., 2018; Deschoenmaeker *et al*., 2019), these three *m*-type Trxs may have independent roles. As shown in Fig. 3B, the amount of Trx-*m*1 protein was markedly higher than that of others in *Anabaena* 7120, implying that Trx-*m*1 regulates numerous target proteins under normal growth conditions. Proteome analysis showing that the disruption of *trxM1* resulted in a remarkable change in the protein expression levels in *Anabaena* 7120 supports this idea (Deschoenmaeker *et al.*, 2018, 2019). A previous report showed that the protein expression of Trx-*m*2 is regulated by the redox-sensing transcriptional regulator RexT and is repressed under reducing conditions (Ehira and Ohmori, 2012). Moreover, the transcript level of *trxM2* is reported to be increased by oxidative stress or nitrogen deprivation (Ehira and Ohmori, 2006). Therefore, Trx-*m*2 appears to be important for the regulation of target proteins under oxidative stress conditions or at the early stage of heterocyst development. Because the *E*_m_ value of Trx-*m*3 was more negative than those of Trx-*m*1 and Trx-*m*2 (Fig. 2B, C), Trx-*m*3 may have the potential to reduce target proteins that have more negative *E*_m_ values.

Alr2205 was reduced by NTR or NTRC, but not by FTR (Fig. 1A, B). Because of its atypical active site YCPSC, Alr2205 is considered not to be reduced by FTR. Alr2205 is encoded downstream of NTR (Alr2204). In addition, two genes encoding Trx-like proteins harboring the WCXXC motif are located upstream of NTR. This series of genes is conserved in several cyanobacteria. However, related species of *Anabaena* 7120 such as *Anabaena variabilis* ATCC 29413 and *N. punctiforme* ATCC 29133 lack these genes. Therefore, genes for NTR and Alr2205 are considered not to be essential for heterocyst-forming, nitrogen-fixing cyanobacteria. These genes may be pseudogenes because their gene products have never been reported, although some transcripts have been detected (Ehira and Ohmori, 2006, 2012).

All Trx proteins examined were reduced only by FTR, except for Alr2205 (Fig. 1A, B). NTRC is an efficient electron donor for the antioxidant enzyme 2-Cys peroxiredoxin in *Anabaena* 7120 (Mihara *et al*., 2017). Although NTRC acts as an electron donor for Trx-*z* in Arabidopsis (Yoshida and Hisabori, 2016), the NTRC-dependent system is considered to function separately from the Trx-dependent system in cyanobacteria. FTR is widely conserved among cyanobacteria, but not observed in *Gloeobacter violaceus* and *Prochlorococcus* species (Florencio *et al*., 2006). Instead, these species have DTR (Buey *et al*., 2017). DTR shows high structural similarity with NTR, but is not reduced by NADPH (Buey *et al*., 2017). Because a recent study showed that Fd-dependent flavin Trx reductase is the closest structural homolog of DTR, DTR is considered to be reduced by Fd (Buey *et al*., 2018). On the basis of these findings, we assume that cyanobacteria have FTR or DTR, and this light-dependent regulatory system may function even in open-ocean cyanobacteria such as *Prochlorococcus* species.

GFP-tagged CP12ΔC was more oxidized in heterocysts than in vegetative cells (Figs 5C, 6), implying that the Trx-dependent redox regulation system does not function in heterocysts. The lack of PSII and limited protein expression of Trx-*m*1 and FTR may affect the reduction level of the Trx target proteins in heterocysts. In plant chloroplasts, it was reported that the reduction of Trxs and their target proteins was restricted by the inhibition of PSII using dichlorophenyl-dimethyl urea (Yoshida *et al*., 2014). Although Fd is reduced by PSI and other metabolic enzymes such as pyruvate ferredoxin oxidoreductase in heterocysts, the lack of PSII is considered to greatly affect the reduction level of Trxs and their target proteins. Our western blotting results indicate that the amount of Trx-*m*1 protein is decreased in heterocysts (Fig. 7B). The transcription of *trxM1* is considered to be co-regulated by NtcA and FurA in *Anabaena* 7120 (Lopez-Gomollon *et al*., 2007). NtcA acts as an activator and a repressor, and plays an important role in heterocyst development. Because putative NtcA-binding sites are located near the putative transcriptional initiation sites of *trxM1*, transcription of these genes might be repressed similarly to that of *rbcL* by NtcA in heterocysts (Lopez-Gomollon *et al*., 2007; Mitschke *et al*., 2011). Moreover, a previous study showed that the transcription of Trx and FTR genes is regulated by photosynthetic electron transport in *Synechocystis* sp. PCC 6803 (Navarro *et al*., 2000; Perez-Perez *et al*., 2009). The lack of PSII might affect Trx gene transcription in heterocysts.

In the case of the non-nitrogen-fixing cyanobacteria *Synechocystis* sp. PCC 6803 and *Synechococcus* sp. PCC 7002, the transcription levels of *zwf*, genes encoding G6PDH, and *opcA* were reported to be increased under nitrogen-depleted conditions (Osanai *et al*., 2006; Shimmori *et al*., 2018). Moreover, G6PDH activity was reported to be increased after nitrogen deprivation in *Synechococcus* sp. PCC 7002 (Shimmori *et al*., 2018). Furthermore, cell viability on the *opcA* knockout of *Synechococcus* sp. PCC 7002 was reported to be lower than that of the wild type in the absence of combined nitrogen (Shimmori *et al*., 2018). These reports imply that the OPPP is activated under light conditions and under nitrogen-depleted conditions, even in non-nitrogen-fixing cyanobacteria. Because phycobiliproteins are degraded and PSII subunits are decreased under nitrogen-depleted conditions in these cyanobacteria, OpcA may be partially oxidized; in this way, G6PDH maintains its activity even under light conditions.

**Fig. 8.**
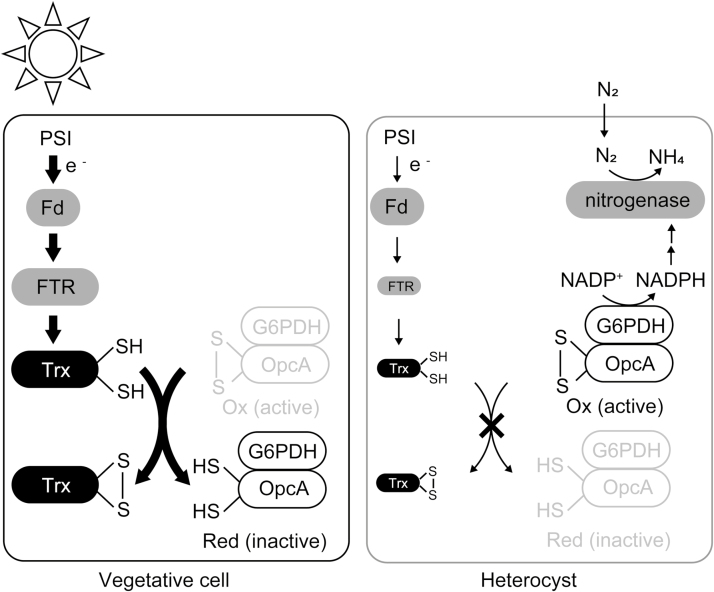
Proposed model of redox cascades in vegetative cells and heterocysts. Our results showed that Trx target proteins are reduced in vegetative cells but oxidized in heterocysts under light conditions. The differences in photosynthetic electron flow and protein expression levels of redox cascade components may affect their redox state.

The present work shows that Trxs are reduced in a light-dependent manner but their target proteins are partly oxidized in heterocysts, even under light conditions, possibly due to the lack of PSII and limited protein expression of Trx-*m*1 and FTR (Fig. 8). Taking these findings together, the redox regulation system is altered in heterocysts; in this way, the redox states of Trx target proteins in *Anabaena* 7120 are affected by not only light conditions but also nitrogen conditions. This light-independent regulation must be important for the performance of both photosynthesis and nitrogen fixation under light conditions.

## Supplementary data

Supplementary data are available at *JXB* online.

**Fig. S1.** Phylogenetic tree of the Trxs in *Anabaena* (Anab), *Synechocystis* sp. PCC 6803 (Syn), and *Arabidopsis thaliana* (At).

**Fig. S2.** NADPH-dependent DTNB reduction activity of NTR.

**Fig. S3.** The entire blotting image of Fig. 3A.

**Fig. S4.** The specificity of anti-Trx-*m*, Trx-*m*3, Trx-*x*, Trx-*y*, and NTRC antibodies.

erz561_suppl_Supplementary_Figures_S1-S4

## Abbreviations:

AMS: 4-acetamido-4-maleimidylstilbene-2,2-disulfonate
*Anabaena* 7120: *Anabaena* sp. PCC 7120
DTNB: 5,5'-dithiobis (2-nitrobenzoic acid)
DTR: deeply rooted bacterial thioredoxin reductase
*E*_m_: midpoint redox potential
FNR: ferredoxin-NADP^+^ reductase
FTR: ferredoxin-thioredoxin reductase
FTR-C: ferredoxin-thioredoxin reductase catalytic subunit
FTR-V: ferredoxin-thioredoxin reductase variable subunit
Fd: ferredoxin
GFP: green fluorescent protein
G6PDH: glucose 6-phosphate dehydrogenase
NTR: NADPH-thioredoxin reductase
NTRC: NADPH-thioredoxin reductase C
OPPP: oxidative pentose phosphate pathway
TCA: trichloroacetic acid
Trx: thioredoxin
