# Supplementary material for: Thioredoxin targets are regulated in heterocysts of cyanobacterium Anabaena sp. PCC 7120 in a light-independent manner
Source: J Exp Bot. 2019 Dec 21;71(6):2018–27. doi: 10.1093/jxb/erz561 (PMC7242069; doi:10.1093/jxb/erz561)
Supplement: erz561_suppl_Supplementary_Figures_S1-S4 [file erz561_suppl_supplementary_figures_s1-s4.pdf]

Supplementary Figures

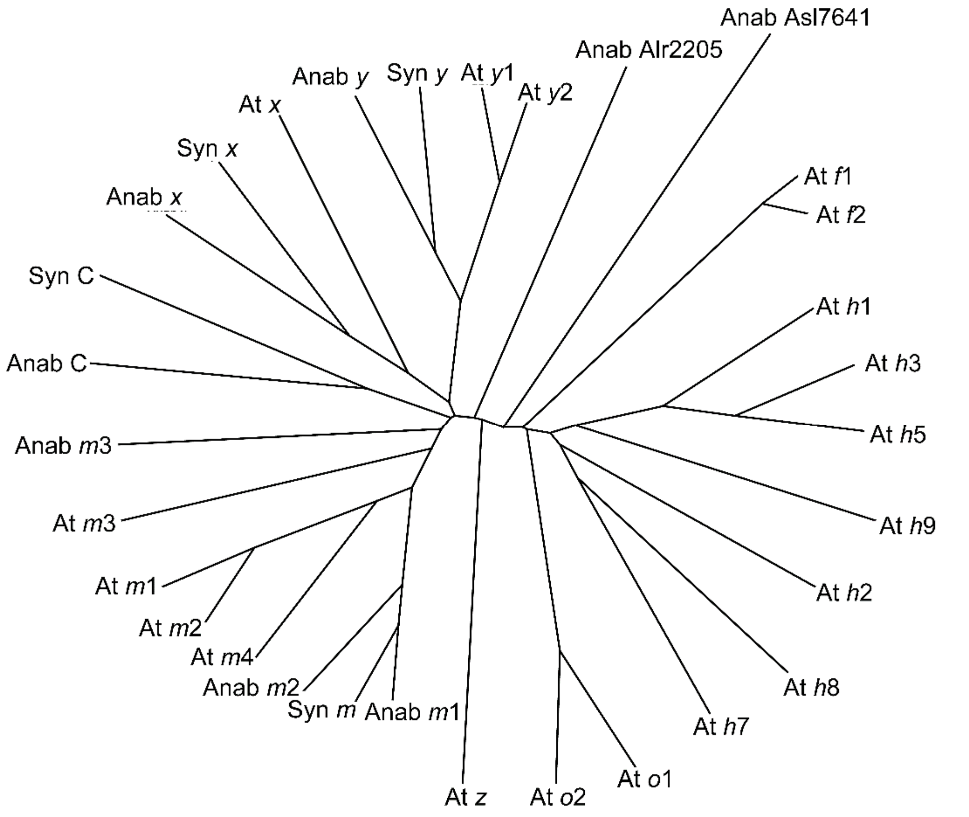

Figure S1 Phylogenetic tree of the Trxs in *Anabaena* (Anab), *Synechocystis* sp. PCC 6803 (Syn) and *Arabidopsis thaliana* (At). The unrooted tree was constructed using ClustalW and written using PHYLIP ver. 3.69. The accession numbers of *Anabaena* Trxs are *m*1, alr0052; *m*2, all1866; *m*3, all2367; *x*, all2341; *y*, all1893; C, alr3955. The accession numbers of *Synechocystis* Trx are *m*, slr0623; *x*, slr1139; *y*, alr0233; C, sll1057. The accession number of *Arabidopsis* Trxs are *f*1, At3g02730; *f*2, At5g16400; *m*1, At1g03680; *m*2, At4g03520; *m*3, At2g15570; *m*4, At3g15360; *x*, At1g50320; *y*1, At1g76760; *y*2, At1g43560; *z*, At3g06730; *h*1, At3g51030; *h*2, At5g39950; *h*3, At5g42980; *h*5, At1g45145; *h*7, At1g59730; *h*8, At1g69880; *h*9, At3g08710; *o*1, At2g35010; *o*2, At1g31020.

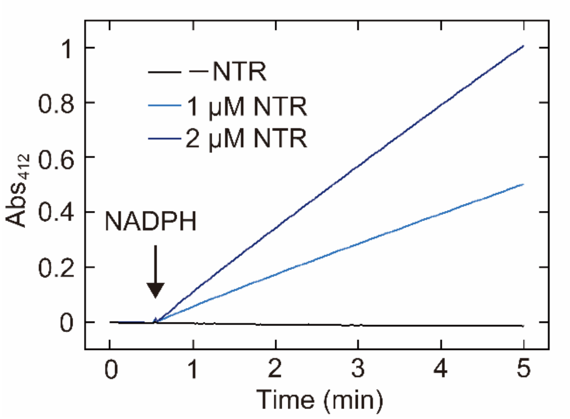

Figure S2 NADPH-dependent DTNB reduction activity of NTR. NTR (1 or 2 μM) was incubated in 50 mM Tris-HCl (pH 7.5) buffer containing 50 mM NaCl, 1mM EDTA and 5 mM DTNB for 30 sec at 30°C. The reaction was started by adding 0.2 mM NADPH. DTNB reduction activity was measured by monitoring the absorbance at 412 nm.

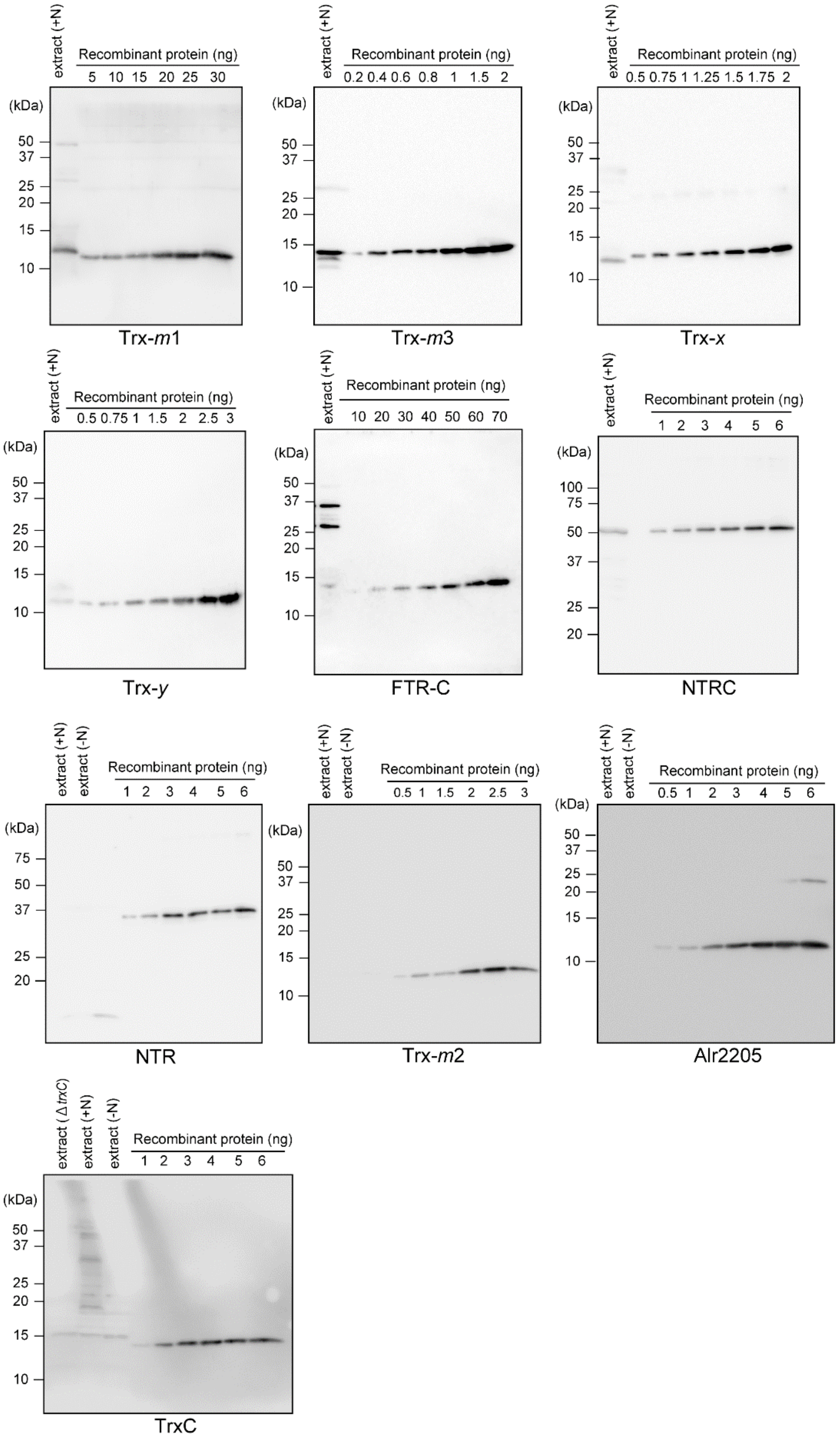

Figure S3 The entire blotting image of Figure 3A.

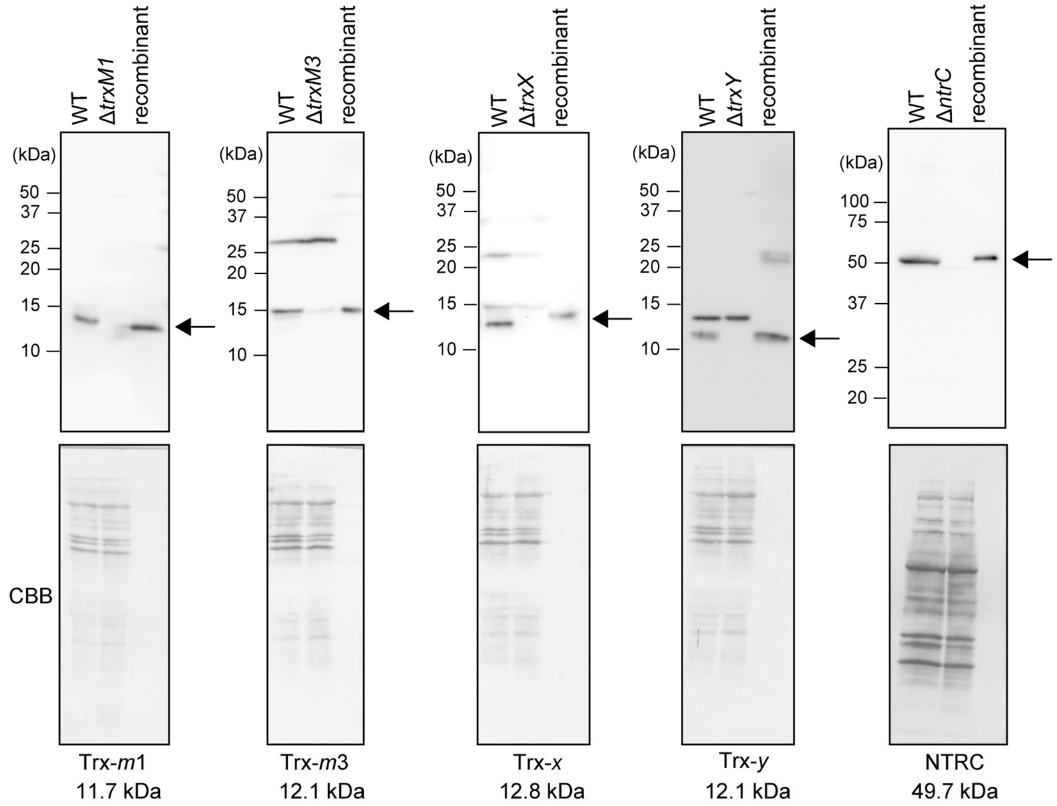

Figure S4 The specificity of anti-Trx-*m*, Trx-*m*3, Trx-*x*, Trx-*y* and NTRC antibodies. The recombinant proteins and proteins extracted from *Anabaena* or its Trx knockout mutants grown in the presence of nitrate were subjected to SDS-PAGE and detected by immunoblotting. The bands corresponding to desired proteins were indicated by arrows.
